# Not All That Glitters Is Gold: The Paradox of CO-dependent Hydrogenogenesis in *Parageobacillus thermoglucosidasius*

**DOI:** 10.3389/fmicb.2021.784652

**Published:** 2021-12-09

**Authors:** Habibu Aliyu, Pieter de Maayer, Anke Neumann

**Affiliations:** ^1^Institute of Process Engineering in Life Science 2 – Technical Biology, Karlsruhe Institute of Technology, Karlsruhe, Germany; ^2^School of Molecular and Cell Biology, Faculty of Science, University of the Witwatersrand, Johannesburg, South Africa

**Keywords:** carbon monoxide, catechol degradation, genomic loci, nickel uptake, hydrogen production, *P. thermoglucosidasius*, water gas shift reaction

## Abstract

The thermophilic bacterium *Parageobacillus thermoglucosidasius* has recently gained interest due to its ability to catalyze the water gas shift reaction, where the oxidation of carbon monoxide (CO) is linked to the evolution of hydrogen (H_2_) gas. This phenotype is largely predictable based on the presence of a genomic region coding for a carbon monoxide dehydrogenase (CODH—Coo) and hydrogen evolving hydrogenase (Phc). In this work, seven previously uncharacterized strains were cultivated under 50% CO and 50% air atmosphere. Despite the presence of the *coo*—*phc* genes in all seven strains, only one strain, Kp1013, oxidizes CO and yields H_2_. The genomes of the H_2_ producing strains contain unique genomic regions that code for proteins involved in nickel transport and the detoxification of catechol, a by-product of a siderophore-mediated iron acquisition system. Combined, the presence of these genomic regions could potentially drive biological water gas shift (WGS) reaction in *P. thermoglucosidasius*.

## Introduction

The genus *Parageobacillus* comprises a phylogenetically coherent group of thermophilic (growth T*^opt^* range of 50–65°C) and facultative anaerobes in the family *Bacillaceae* (Suzuki et al., 1983; Aliyu et al., 2016, 2018; Oren and Garrity, 2019). Although they share many features, including growth temperature requirement (obligate thermophiles) with their closest relatives in the genus *Geobacillus*, *Parageobacillus* species are distinguished by a characteristic low genomic G + C content and a distinct phylogenetic clustering among several other phylogenomic features (Aliyu et al., 2016, 2018). At present, the genus comprises six species with taxonomic standing (Najar et al., 2020), including the type species of the genus, *Parageobacillus thermoglucosidasius* (Suzuki et al., 1983; Aliyu et al., 2016).

A search of the NCBI database (21.05.2021), revealed 28 (excluding anomalous) *Parageobacillus* genome assemblies, including 16 (∼57%) assemblies of *P. thermoglucosidasius* strains isolated from distinct sources. The bias toward sequencing numerous strains could be associated with a greater interest in the biotechnological potential of this species. Due to its catabolic versatility (Hussein et al., 2015), *P. thermoglucosidasius* grows optimally on a range of carbohydrates, ranging from the complex hemicellulosic substrates (e.g., xylan) to sugar monomers (e.g., hexoses and pentoses), making it a suitable candidate for biomass degradation in ethanologenesis workstreams (De Maayer et al., 2014; Zeigler, 2014; Brumm et al., 2015a). Thus, the organism could be explored as a source of thermostable enzymes, including carbohydrate active enzymes such as amylases and xylanases, lipases and proteases (Zhu et al., 2007; Abdel-Fattah and Gaballa, 2008; Mok et al., 2013; Huang et al., 2014; Jiang et al., 2015). The combination of the above properties has endeared the development of several strains for various biotechnological applications (Hussein et al., 2015; Wada and Suzuki, 2020).

Prediction of hydrogen production capacity, albeit based on the assumption of the Wood–Ljungdahl pathway in the genome of *P. thermoglucosidasius* Y4.1MC1 (Brumm et al., 2015b), prompted further investigations in members of this species. Various studies have reported the production of hydrogen (H_2_) *via* carbon monoxide (CO) oxidation by *P. thermoglucosidasius* strains (Table 1), thereby expanding their metabolic versatility to include gaseous substrates and the range of potential biotechnological and industrial applications for this organism. Analyses of genomes of *P. thermoglucosidasius* strains showed that they harbor a unique fifteen gene genomic region, comprising the genes *cooCSF* and *phcABCDEFGHIJKL* which code for a CODH and Ni-Fe group 4a hydrogenase, respectively (Mohr et al., 2018a,b). CODH catalyzes the oxidation of carbon monoxide (CO) to CO_2_ and electrons, while the Ni-Fe hydrogenase catalyzes the reduction of protons from H_2_O (Henstra et al., 2007; Antonopoulou et al., 2011; Schoelmerich and Müller, 2019). Combined, these enzymes catalyze the water gas shift (WGS) reaction (CO + H_2_O → CO_2_ + H_2_ ΔG^*O*Δ^ = −20 kJ/mol CO), which forms the basis for the observed H_2_ evolution in the presence of CO (Mohr et al., 2018a). The demonstration of this strictly anaerobic process, a first among facultative anaerobes (Mohr et al., 2018a,b), may provide additional insight into genomic evolution and dynamics of prokaryotic phenotypes.

**TABLE 1:** Features of *Parageobacillus thermoglucosidasius* strains included in this study.

Strain	Isolation source	Genome accession	Assembly size	# proteins	# genes unique to H_2_ producers	WGS references
TG4	marine sediment	GCF_003865195.2	3,948,523	3909	20	Adachi et al., 2020
Y4.1MC1	Hot spring	GCF_000166075.1	3,911,947	3842	0	This study
DSM 2542^T^	Soil	GCF_001295365.1	3,873,116	3889	20	Mohr et al., 2018a
DSM 21625	Flax plant bast fiber	GCF_014218665.1	4,006,039	3939	0	Mohr et al., 2018b
C56-YS93	Hot spring	GCF_000178395.2	3,993,793	3966	0	This study
M10EXG	Compost	IMG_2501416905	3,773,252	3727	0	This study
DSM 6285	River sediment	GCF_014218645.1	3,967,726	3888	20	Mohr et al., 2018b

Recent mutagenesis studies (Adachi et al., 2020), involving the knockout of the CODH and hydrogenases loci, individually and collectively, has conclusively shown that the predicted CODH and hydrogenase genes (above) code for the enzymes that catalyze WGS in *P. thermoglucosidasius*. While the above studies have confirmed the functional role of the *coo*—*phc* locus, central questions on the regulation of the process remain unclear. One outstanding question relates to the observation that one strain *P. thermoglucosidasius* DSM 21625 isolated from plant fiber lacked WGS reaction function despite harboring the CODH-hydrogenase gene locus (Mohr et al., 2018b). The absence of the phenotype was attributed, partly, to the presence of two deletions, indel 1 (17 nt) and indel 2 (22 nt) 115 nt upstream of *cooC* and in the region between *cooC* and *cooS*, respectively. These indels were speculated to include regulatory signatures that potentially prevent the expression of the locus (Mohr et al., 2018b). Other genomic differences, including the occurrence of several non-synonymous substitutions within the *coo*—*phc* locus as well as the presence of unique gene complements of unknown function in both *P. thermoglucosidasius* DSM 21625 and the hydrogenogenic strains, have been suggested to play a potential role in shaping the hydrogenogenic phenotype (Mohr et al., 2018b).

The presence of the co-localized genes coding for the CODH and hydrogen evolving Ni-Fe hydrogenase Phc, and the ability of strains that have been characterized to utilize CO and produce H_2_ have, so far, led to the prediction of the latter phenotype in all studied *P. thermoglucosidasius* strains harboring these genes. For instance, in a recent review (Fukuyama et al., 2020), several strains, including the type species *P. thermoglucosidasius* DSM 2542^T^ and Y4.1MC1 have been listed as potentially CO oxidizing and hydrogenogenic *P. thermoglucosidasius* strains. However, “not all that glitters is gold,” previous comparative studies have revealed that despite having the *cooCSF-phcA-L* genes as observed in three other *P. thermoglucosidasius* strains (DSM 2542^T^, DSM 2543, and DSM 6285), DSM 21625 is incapable of catalyzing the WGS reaction (Mohr et al., 2018b). Therefore, to evaluate the extent of this seemingly interesting evolutionary pattern, that is the presence of the locus and absence of the phenotype among *P. thermoglucosidasius* strains, we characterized relevant genomic fragments and performed fermentation experiments among selected and diverse *P. thermoglucosidasius* strains (Table 1).

## Materials and Methods

### Strain, Media, and Growth Condition

Seven *P. thermoglucosidasius*, C56-YS93, Kp1012, Kp1013, Kp1019, M10EXG, M5EXG and Y4.1MC1, were obtained from the *Bacillus* genetic stock center (BGSC), United States, and stored as glycerol stock in a −80°C freezer. Each strain was revived *via* a 14 h pre-culture in a shake flask containing 20 mL modified Luria–Bertani (mLB) medium (10 g/L tryptone, 5 g/L yeast extract, 5 g/L NaCl, 1.25 mL/L of 10 g/L NaOH and 1 mL/L of each of the filter-sterilized stock solutions 1.05 M nitrilotriacetic acid, 0.59 M MgSO_4_⋅7H_2_O, 0.91 M CaCl_2_⋅2H_2_O and 0.04 M FeSO_4_⋅7H_2_O) incubated in a Thermotron (Infors, Switzerland) set at 120 rpm and 60°C. The same medium and incubation conditions were used for the main experiments. The strains were cultivated for 72 h in 250 mL serum bottles sealed with a rubber stopper (top: 18 mm, bottom 14 mm and height 20 mm, Rotilabo^®^, Carl Roth, Karlsruhe, Germany), containing 50 mL mLB and the 200 mL of headspace atmosphere reconstituted to a 50:50 ratio of CO to air. A second experiment was conducted under above condition using the ammonium salts medium ASM, pH 6.8 (Mohr et al., 2018b,^2019^). ASM comprises 8.7 mM citric acid, 20.2 mM MgSO_4_, 10 mM K_2_SO_4_, 22.6 mM NaH_2_PO_4_, 0.8 mM CaCl_2_, 25 mM (NH_4_)_2_SO_4_, 4.16 mM glucose and trace elements (0.012 mM H_2_SO_4_, 0.002 mM CuSO_4_, 0.004 mM CoSO_4_, 0.010 mM ZnSO_4_, 0.046 mM FeSO_4_, 0.006 mM NiSO_4_, 0.018 mM MnSO_4_ and 0.002 mM H_3_BO_3_).

At each sampling point ∼3 mL gas samples were collected and analyzed using a 300 Micro GC gas analyser (Inficon, Switzerland) connected with 10 m Molsieve (channel 1)and 10 m PoraPLOT Q (channel 2) columns. Channel 1 detects CO, H_2_ and N_2_ with Ar used as carrier gas while channel 2 detects CO_2_ with He used as carrier gas. Both channels are equipped with thermal conductivity detector and the column temperature was set at 80°C. Final gas composition was calculated using the ideal gas law as previously described (Mohr et al., 2018b,^2019^). For each bottle, 1 mL liquid sample was analyzed for growth (OD_600_) and pH, using Ultrospec 1100 pro spectrophotometer (Amersham Biosciences, United States) and Profilab pH 597 (Xylem Analytics, Germany), respectively.

### Phylogenetic Validation and Analyses

Genomic DNA was extracted from the above seven strains and DSM 21625, DSM 2542^T^, and DSM 6285 using Quick-DNA Fungal/Bacterial Miniprep Kit (Zymo Research). Partial *gyrA*, *recN* and *rpoB*, gene fragments of each strain were amplified by PCR using OneTaq Hot Start Quick-Load 2X Master Mix, Standard Buffer (New England Biolabs) with the primer pairs; *gyrA*f (GCAAAGCGTATGAAACAGG) and *gyrA*r GTTCGACAAAGTCATCTTCG), *recN*f (ACGCTTGTCGATAT TCACG) and *recN*r (CGCTAAGACGGCTTTCAAT), and *rpoB*f (GTTTGCATCCGCTTGTATG) and *rpoBr* (TCTTAAA TGGCGGAACGAG). A phylogenetic tree was constructed based on the concatenated alignments of the above genes using IQ-TREE v1.6.7 with the optimal substitution model determined through model test and ultrafast support (UFBoot; *n* = 1,000 replicates) (Schmidt et al., 2014). The phylogeny was visualized using MEGA7 (Kumar et al., 2016). Genome-based taxonomic affiliation of the seven compared *P. thermoglucosidasius* genomes (Table 1) was verified using GTDB-Tk v1.7.0 (Chaumeil et al., 2019; Parks et al., 2020). All basic sequence processing and computation of the identity matrix were performed using BioEdit (Hall, 1999).

### Analysis of the *cooCSF-phcA-L* Locus and the Unique Genome Fragments of H_2_ Producing Strains

Prokka v 1.14.6 (Seemann, 2014) was used to annotate the available genomes of seven strains (Table 1) and those of three closely related *Parageobacillus* species.

The *cooCSF-phcA-L* gene loci were mapped against the genomes of the seven strains using SimpleSynteny (Veltri et al., 2016). To identify putative transcription factors binding sites (TFBs) associated with the previously reported deletion fragments within the *coo*—*phc* locus (Mohr et al., 2018b), 749 and 680 bp from -1 position upstream of *cooC* and the region between *cooC* and *cooS*, respectively, which encompass the positions of two deletions were extracted from the genome of *P. thermoglucosidasius* DSM 2542^T^ (used as reference) and analyzed using BPROM (Solovyev and Salamov, 2011) and CNNPromoter_b (Umarov and Solovyev, 2017). Finally, the position of the deletions were amplified by PCR using OneTaq Hot Start Quick-Load 2X Master Mix, Standard Buffer (New England Biolabs) and based on the following primer pairs; I1bf (TGTTCGGCTTTTCTGAGGTT) and I1br (ATGCCATTGTTTGTCCAGGT) and I2bf (CATGGACC TTGCAAACAGTG) and I2bR (CGGGAATTGAACACTT GACC). I1bf/r and I2bf/r primer pairs amplify 622 and 609 bp encompassing the positions of the first and second deletions, respectively.

The predicted proteomes of the seven *P. thermoglucosidasius* strains were compared using Roary (Page et al., 2015) with minimum amino acid similarity threshold set at 90%. The unique proteome compliment of the hydrogen producing strains was identified using scoary (Brynildsrud et al., 2016). The predicted proteins of the compared strains were functionally annotated using BlastKOALA (Kanehisa et al., 2016) and KofamScan (Aramaki et al., 2019) (as implemented in https://github.com/takaram/kofam_scan), eggNOG v5.0 (Huerta-Cepas et al., 2018) and NCBI conserved domain (CD) search (Lu et al., 2019). KEGG Mapper (Kanehisa and Sato, 2020) was used to determine metabolic pathways associated with the unique genomic loci of the hydrogenogenic *P. thermoglucosidasius* strains.

Presence or absence orthologs of the unique protein sets along with those within the same genomic regions were confirmed using BlastP, BlastN and tBlastN analyses against the genome sequences. The loci were subsequently mapped against the respective genomic regions of the compared genomes using SimpleSynteny (Veltri et al., 2016).

*Parageobacillus thermoglucosidasius* DSM 6285 expression trajectories (Aliyu et al., 2020) computed using DP_GP_cluster (McDowell et al., 2018) were used to determine the profiles of the unique genes associated with two relevant genomic regions over the course of the organism’s growth under an initial gas composition of 50:50 CO to air.

## Results

### Taxonomic Evaluation

**FIGURE 1:**
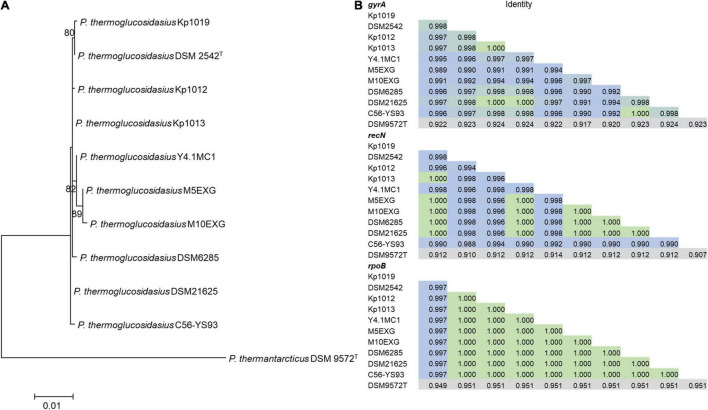
Phylogenetic analysis of ten *P. thermoglucosidasius* strains included in this study with *P. thermantarcticus* DSM 9572^T^ used as outgroup. **(A)** Maximum likelihood tree of concatenated nucleotide alignment (1,851 characters) of PCR amplified *gyrA, recN* and *rpoB* partial sequences. The ML tree was inferred using IQ-TREE (Schmidt et al., 2014), **(B)** Identity matrices of the individual alignments of gyrA, recN and rpoB.

Phylogenetic analysis based on the concatenated alignment of PCR amplified partial sequences of *gyrA*, *recN* and *rpoB* (Figure 1) showed that all evaluated isolates obtained from the BGSC clustered with the type strain *P. thermoglucosidasius* DSM 2542^T^. The phylogeny and sequence identity values suggest that all ten isolates subsequently evaluated in this work are *P. thermoglucosidasius* strains (Figure 1A). Evaluation of the trimmed alignments of *gyrA* (852 nt), *recN* (527 nt) and *rpoB* (472 nt) revealed that these fragments shared an identity range of 98.8–100% among the *P. thermoglucosidasius*. By contrast, these gene fragments from *P. thermoglucosidasius* strains shared 92.0, 91.0–95.0% identity for *gyrA*, *recN* and *rpoB*, respectively, with those from *P. thermantarcticus* DSM 9572^T^ (Figure 1B). Genome Taxonomy Database Toolkit (GTDB-Tk) evaluation of the seven publicly available *P. thermoglucosidasius* genomes included in this work (Table 1) also confirms their affiliation to *P. thermoglucosidasius* (Supplementary Table 1).

### Water Gas Shift Reaction and H_2_ Production Among *Parageobacillus thermoglucosidasius* Strains

**FIGURE 2:**
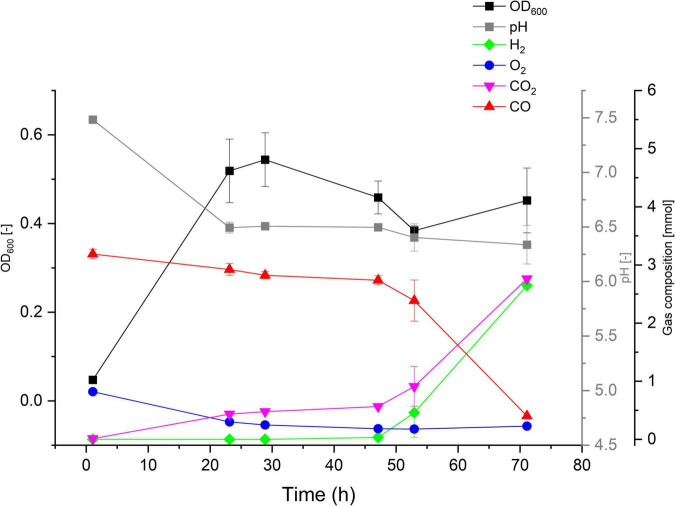
Gas composition and growth of *P. thermoglucosidasius* Kp1013 cultivated under 50% carbon monoxide and 50% air in 250 ml serum bottles containing 50 ml of mLB medium.

The ability (or lack thereof) of *P. thermoglucosidasius* DSM 2542^T^, DSM 6285, and DSM 21625 to catalyze the WGS reaction has been reported previously (Mohr et al., 2018b). To evaluate the hydrogenogenic capacity of seven *P. thermoglucosidasius* strains, for which no data on hydrogenogenesis is available, were cultivated in 250 ml serum bottles containing 50 ml mLB and initial headspace gas composition of 50% CO and 50% air for a duration of 70 h. Our analysis showed that, of the seven strains, only Kp1013 could utilize CO to yield H_2_ (Figure 2). The strain showed growth dynamics under 50% CO similar to *P. thermoglucosidasius* DSM 2542^T^ and DSM 6285 (Mohr et al., 2018b), with initial aerobic growth to a maximum OD_600_ value of ∼0.54 after 29 h cultivation. This was followed by a prolonged lag phase prior to the commencement of the WGS reaction, during which the OD_600_ value dropped to a minimum of ∼0.38 (Figure 2). The first indication of WGS was recorded around 47 h (0.03 mmol H_2_) and the maximum of 2.7 mmol H_2_ was observed at 70 h. This corresponds to the observed CO consumption from an initial value of 3.19–0.40 mmol (Figure 2).

By contrast, gas composition analysis over the duration of 70 h revealed that *P. thermoglucosidasius* C56-YS93, M5EXG, M10EXG, Kp1012, Kp1019 and Y4.1MC1 did not utilize CO and no H_2_ was detected throughout the cultivation period under the set conditions (Supplementary Figure 1). In all instances, the strain showed rapid initial aerobic growth (based on OD_600_) in the presence of CO, which suggests a general tolerance to the gas by *P. thermoglucosidasius* strains. However, the absorbance (OD_600_) values of the strains steadily declined after O_2_ depletion. Compared to *P. thermoglucosidasius* Kp1013 (Figure 2), four of the non-hydrogenogenic strains, *P. thermoglucosidasius* C56-YS93, M5EXG, M10EXG and Kp1012 (Supplementary Figures 1A–D) showed higher absorbance of ∼ 0.58, 0.76, 0.83, and 0.64, respectively, in the aerobic growth phase. Conversely, *P. thermoglucosidasius* Kp1019 showed the least growth among the cultivated organisms with a maximum OD_600_ value of ∼0.31 (Supplementary Figure 1E).

Although, WGR reaction was initially demonstrated using the complex mLB medium (Mohr et al., 2018a,b), the strains were screened in ASM medium for a duration of 337 h under the conditions described above. As observed with the mLB medium, all *P. thermoglucosidasius* strains were capable of growth in the presence of CO (Supplementary Figure 2). However, only Kp1013 consumed CO and produced H2 (Supplementary Figure 2A), thereby providing further support for the absence of WGS reaction phenotype among six additional *P. thermoglucosidasius* strains under 50% CO and 50% air. It is noteworthy, however, that the actual maximum OD_600_ values during the initial aerobic growth phase could not be estimated reliably due to the extended duration of the initial sampling intervals. However, the goal of the study was to ascertain if the investigated strains have the capacity to utilize CO to generate H_2_.

### Conservation of *Parageobacillus thermoglucosidasius cooCSF-phcA-L* Genomic Region

**FIGURE 3:**
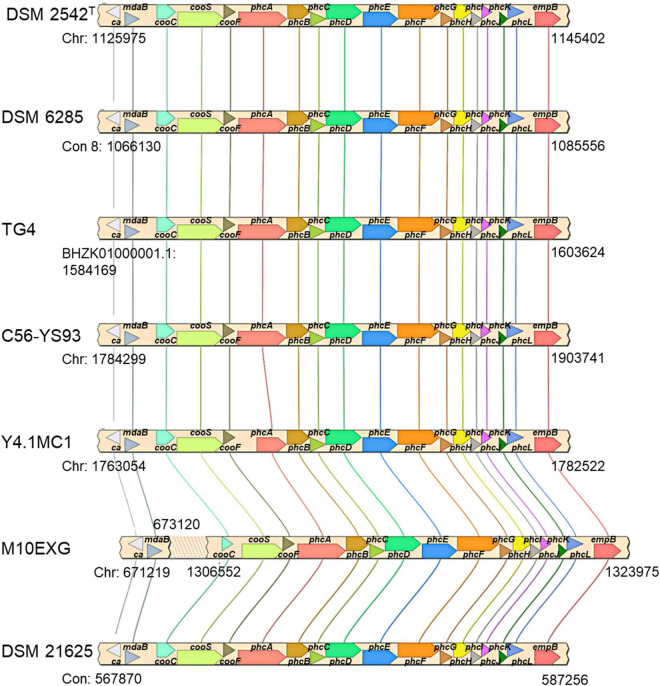
*cooCSF-phcA-L* locus in seven *P. thermoglucosidasius* genomes visualized using SimpleSynteny. Only strains DSM 2542^T^, DSM 6285 and TG4 have been shown to produce hydrogen *via* CO oxidation.

The co-localization of the *cooCSF* and *phcA-L* genes is a unique feature of *P. thermoglucosidasius* species (Mohr et al., 2018b). Further, the presence of these genes suggests that all *P. thermoglucosidasius* spp. have the potential to catalyze WGS reaction. Analysis of the available *P. thermoglucosidasius* genome sequences showed that six strains harbor orthologs of the *cooCSF* and *phcA-L* genes flanked by genes of carbonic anhydrase and NAD(P)H oxidoreductase (*can* and *mdaB*) at the 5′ end and lysine 2,3-aminomutase (*empB*) at the 3′ end (Figure 3). In the genome of the seventh strain, *P. thermoglucosidasius* M10EXG, *mdaB* and *can* occur −634,343 bp upstream of *cooS*. The conservation of this genomic region among most of the strain suggests a possible misassembled genome or unique chromosomal rearrangement for the latter strain.

**FIGURE 4:**
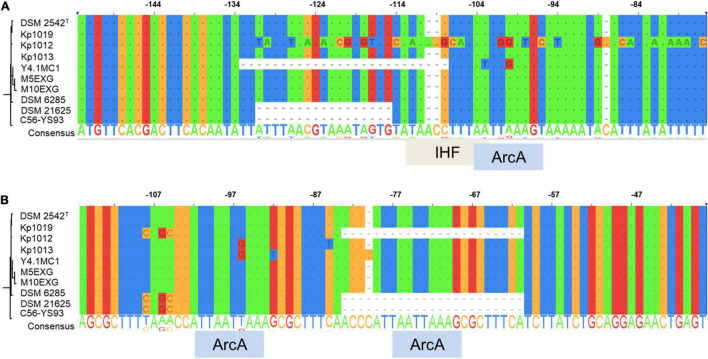
Alignment of PCR amplified deletions adjacent to the *cooC* reading frame among ten *P. thermoglucosidasius* strains characterized for hydrogenogenesis. **(A)** Indel 1 upstream of *cooC* and associated transcription factor binding sites (TFBs), **(B)** Indel 2 between *cooC* and *cooS* and associated TFBs. ArcA and IHF are the aerobic respiration control protein and integration host factor, respectively.

The occurrence of indels 1 and 2 was hypothesized to play a role in the lack of WGS phenotype (Mohr et al., 2018b). The genomic regions encompassing these deletions were amplified in ten *P. thermoglucosidasius* strains, including DSM 21625, using PCR (Figure 4). Our analysis showed that the genomes of two additional strains, C56-YS93 and Y4.1MC1 contain the 17 nt indel 1 present in *P. thermoglucosidasius* DSM 21625, while in Kp1012 the adjacent genomic region incorporates distinct genetic elements in place of indel 1 (Figure 4A). The 22 nt indel 2 observed in *P. thermoglucosidasius* DSM 21625 is further present between the *cooS* and *cooF* genes on the genomes of *P. thermoglucosidasius* Kp1012 and C56-YS93 (Figure 4B). BPROM (Solovyev and Salamov, 2011) prediction of promoters and transcription factor binding sites (TFBs) within the genomic regions containing the above deletions revealed that no TFBs occur within indel 1 (Figure 4A and Table 2). By contrast, one TFB, associated with ArcA was predicted within indel 2 (Figure 4B). However, the predicted ArcA binding site occur in multiple locations within the analyzed sequences (Table 2). TFB screening using CNNPromoter_b (Umarov and Solovyev, 2017) trained on both *Escherichia coli and B. subtilis*, however, predicted only the TFBs of proteins coded by *ihf*, *phoB* and *arcA* in the genomic region of indel 1 and *arcA* for indel 2 (data not shown). Additionally, TFBs for the genes of *irp* (ATTTTTTT) and *lexA* (TTTTTTTA) were predicted in the vicinity of indel 1 and *tyrR* (TGTAATTT) up and downstream of indel 2 genomic regions. However, none of the latter predictions is associated with the deleted fragments.

**TABLE 2:** Putative promoters and transcription factor binding sites (TFBs) upstream of *cooC* and *cooS*.

Promoter	Position	TBS	−10 box	−35 box	Gene/protein	Scores
1	−75 *cooC*					LDF– 14.4
	−114	TATACTTT			*ihf*	15
	−109	TTTAATTA			*phoB*	11
	−108	TTAATTAA			*arcA*	13
	−107	TAATTAAA			*arcA*	11
	−97	AAAAATAA			*fis*	9
	−95	AAATAATT			*rpoD16*	15
	−88	TTATATTT			*gcvA*	11
	−85	TATTTTTT			*lrp*	11
	−83	TTTTTTAT			*argR*	13
	−82	TTTTTATT			*argR2*	13
	−81	TTTTATTT			*ihf*	13
	−80	TTTATTTT			*argR2*	7
	−72	ACAAAAAA			*ihf*	9
	−66	AATTTGAG			*glpR*	6
2	−54 *cooS*					LDF– 4.87
	−101	TTAATTAA			ArcA	13
	−100	TAATTAAA			ArcA	11
	−79	TTAATTAA			ArcA	13
	−78	TAATTAAA			ArcA	11

*Promoters and TFBs were predicted using BPROM on 749 and 680 bp sequences starting from −1 position upstream of cooC and cooS including the genomic positions of two distinct indels.*

### Hydrogenogenic *Parageobacillus thermoglucosidasius* Harbor Two Unique Genetic Loci

Evaluation of the predicted protein sequences of seven *P. thermoglucosidasius* and related *Parageobacillus* spp., using Roary and Scoary (Page et al., 2015; Brynildsrud et al., 2016) showed that twenty distinct proteins are unique to the three CO oxidizing strains (*P. thermoglucosidasius* DSM 2542^T^, DSM 6285, and TG4) among the analyzed *P. thermoglucosidasius* strains (Supplementary Table 2). Using the *P. thermoglucosidasius* DSM 2542^T^ genome as reference, the analysis showed that fifteen of the above proteins are notably associated with two genomic regions on the chromosome and plasmid 1 (Mohr et al., 2018b), respectively.

**FIGURE 5:**
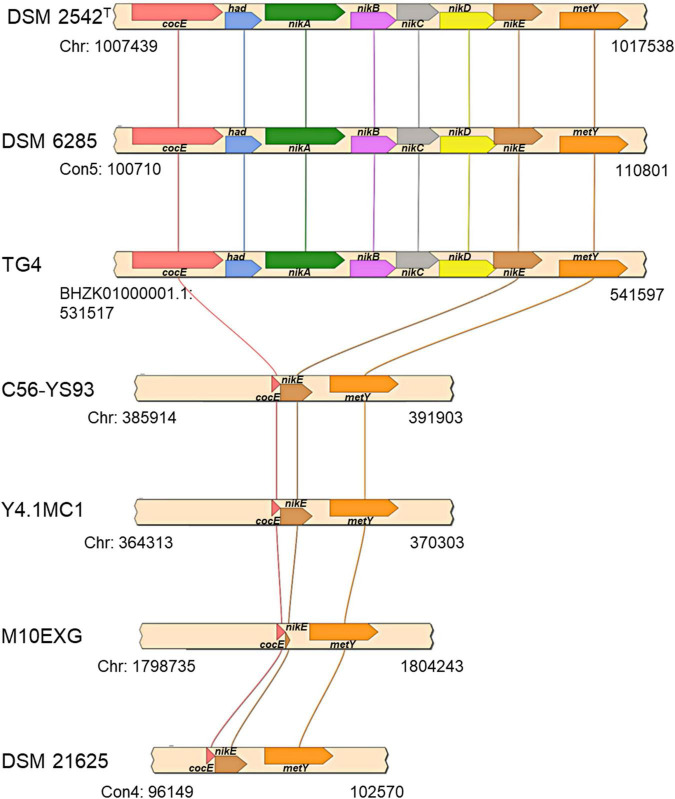
Organization of putative nickel transport locus (HSUL 1) present on hydrogen producing *P. thermoglucosidasius* strains, rendered with aid of SimpleSynteny. *coCE, had, nikA, nikBC, nikDE*, and *metY* code for putative cocaine esterase, putative hydrolase of the HAD superfamily, peptide/nickel transport system substrate-binding, permease, ATP-binding proteins, and *O*-acetylhomoserine (thiol)-lyase, respectively.

The first locus (hydrogenogenic strain unique locus; HSUL 1), located between 1,007,746–1,017,231 bp on the chromosome of *P. thermoglucosidasius* DSM 2542^T^, comprises four genes, *nikABCD*, that code for orthologs of ABC-type dipeptide/oligopeptide/nickel transport system proteins (Eitinger and Mandrand-Berthelot, 2000; Mohr et al., 2018b) flanked by genes coding for a serine esterase and haloacid dehalogenase (HAD) hydrolase (*cocE* and *had*) at the 5′ end and a gene coding for *O*-acetylhomoserine/*O*-acetylserine sulfhydrylase (*metY*) at the 3′ end (Figure 5). The five ABC-type dipeptide/oligopeptide/nickel transport system proteins comprise a substrate-binding protein (NikA), two permeases (NikB and NikC) and two ATP-binding proteins (NikD and NikE) (Figure 5). Similar nickel permease systems have been described in several bacterial taxa (Eitinger and Mandrand-Berthelot, 2000; Hebbeln and Eitinger, 2004). The fifth gene in the HUSL1 locus, *nikE* is shared by all the strains except MX10EXG, where only a 46 nt fragment, matching (100%) the end (position: 885–930) of DSM 2542^T^ *nikE* remains (Figure 5). In addition, MX10EXG and the other strains that are incapable of WGS reaction, harbor only 166 nt fragment of the 1,734 nt *cocE* gene of *P. thermoglucosidasius* DSM 2542^T^ (Figure 5). Combined, the absence of *cocE* and *nikABCD* among the non-hydrogenogenic strains, as well as the presence of only partial copies of *cocE* and *nikE* in *P. thermoglucosidasius* MX10EXG strongly suggest the loss of HSUL 1 among the non-hydrogenogenic strains.

**FIGURE 6:**
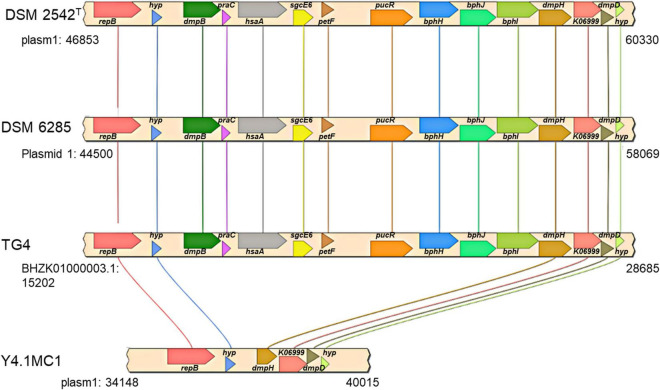
Organization of a plasmid phenol degradation locus (HSUL 2) harboring a ferredoxin (petF) gene unique to hydrogen producing *P. thermoglucosidasius* strains. *repB, dmpB, praC, hsaA, sgcE6, petF, pucR, bphH, bphJ, bphI, dmpH, K06999, dmpD* and *hyp*, which code for initiator replication protein, catechol 2,3-dioxygenase, 4-oxalocrotonate tautomerase, 3-hydroxy-9,10-secoandrosta-1,3,5(10)-triene-9,17-dione monooxygenase, flavin reductase, ferredoxin, purine catabolism regulatory protein, 2-oxopent-4-enoate hydratase, acetaldehyde/propanal dehydrogenase, 4-hydroxy-2-oxovalerate/4-hydroxy-2-oxohexanoate aldolase, 2-oxo-3-hexenedioate decarboxylase, phospholipase/carboxylesterase, 2-hydroxymuconate-semialdehyde hydrolase and proteins of unknown function, respectively.

The second gene set unique to the hydrogenogenic strains occurs on plasmid 1 (position: 47,057–62,595) in *P. thermoglucosidasius* DSM 2542^T^ (Figure 6). The locus (HSUL 2) comprises twelve genes that code for proteins in aromatic hydrocarbon degradation (Ghosal et al., 2016), flanked by two hypothetical proteins (Figure 6). Of these, nine genes occur uniquely among the CO-oxidizing strains while three genes, *dmpD*, “K06999” and *dmpH* are only present in *P. thermoglucosidasius* Y4.MC1 among the non-hydrogenogenic strains. The conservation of the above flanking genes suggests the loss of the genomic region among the non-hydrogenogenic strains.

**FIGURE 7:**
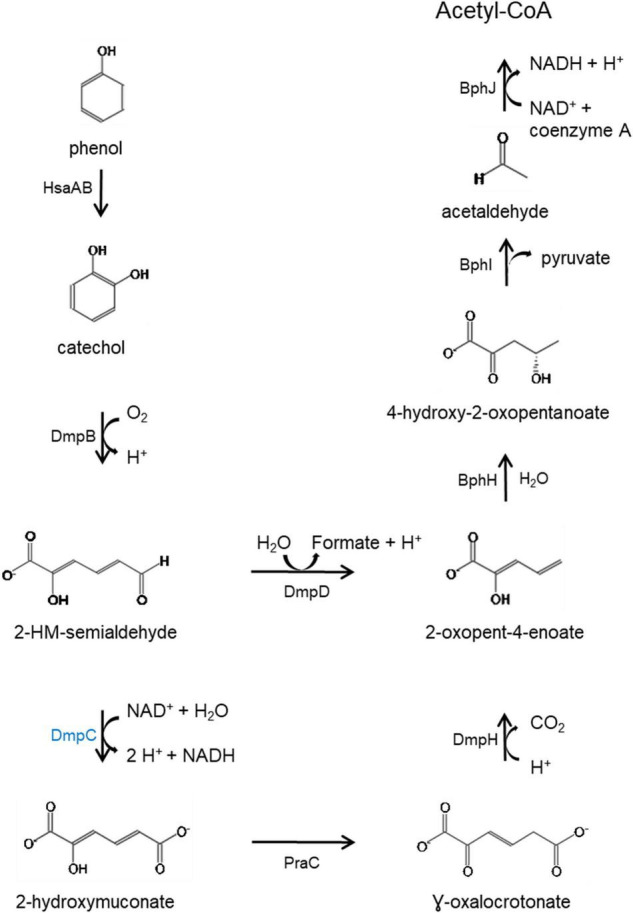
Catabolic pathway for the degradation of phenol *via* meta-cleavage. HsaAB predicted as two component proteins SgcE6 (flavin reductase) and HpaB 4-hydroxyphenylacetate 3-monooxygenase and, SgcE6 (flavin reductase) and HsaA (3-hydroxy-9,10-secoandrosta-1,3,5(10)- triene-9,17-dione monooxygenase) coded by genes in the chromosomal and plasmid phenol degradation loci, respectively. Catechol is catabolized by catechol 2,3-dioxygenase (DmpB), aminomuconate-semialdehyde dehydrogenase (DmpC), 2-hydroxymuconate-semialdehyde hydrolase (DmpD), 4-oxalocrotonate tautomerase (PraC), 2-oxo-3-hexenedioate decarboxylase (DmpH), 2-oxopent-4-enoate/*cis-*2-oxohex-4-enoate hydratase (BphH), 4-hydroxy-2-oxovalerate/4-hydroxy-2-oxohexanoate aldolase (BphI) and acetaldehyde/propanal dehydrogenase (BphJ). Except HsaAB, defined above, all enzymes in black font are coded by gene in both chromosome and plasmid while DmpC, highlighted in blue is coded by the chromosomal gene (*dmpC*). 2-hydroxymuconate-semialdehyde abbreviated as 2-HM-semialdehyde. Meta-cleavage pathway adapted from Pathway Tools v22.0/MetaCyc database (Caspi et al., 2015).

KEGG Mapper (Kanehisa and Sato, 2020) analysis of the predicted proteins coded by genes in the HSUL 2 showed that five genes, *dmpB, bphH, bphJ, bphI*, and *dmpD* are linked to the conversion of catechol into intermediates of central carbon metabolism *via* catechol meta-cleavage (Figure 7). In this pathway (Harayama et al., 1989; Harayama and Rekik, 1990; Fuchs et al., 2011; Suenaga et al., 2014), catechol 2,3-dioxygenase (DmpB) cleaves the aromatic ring of catechol to 2-hydroxymuconate-semialdehyde, which is further hydrolyzed to 2-oxopent-4-enoate by 2-hydroxymuconate-semialdehyde hydrolase (DmpD) (Díaz and Timmis, 1995). 2-oxopent-4-enoate hydratase (BphH) hydrates this product to yield 4-hydroxy-2-oxopentanoate (Harayama et al., 1989; Harayama and Rekik, 1990). Subsequently, 4-hydroxy-2-oxovalerate aldolase (BphI) catalyzes the split of 4-hydroxy-2-oxopentanoate to pyruvate and acetaldehyde (Harayama et al., 1989; Harayama and Rekik, 1990). The latter product is finally oxidized to acetyl-CoA by acetaldehyde dehydrogenase (BphJ). HSUL 2 also harbors *sgcE6* and *hsaA* coding for flavin reductase and 3-hydroxy-9,10-secoandrosta-1,3,5(10)-triene-9,17-dione mono-oxygenase (Figure 6). HsaAB, comprising a reductase and oxygenase, was shown to metabolize 3-hydroxy-9,10-seconandrost-1,3,5(10)-triene-9,17-dione to catechol in *Mycobacterium tuberculosis* (Dresen et al., 2010), suggesting that HSUL 2 encoded SgcE6 and HsaA likely function as a phenol hydroxylase.

**FIGURE 8:**
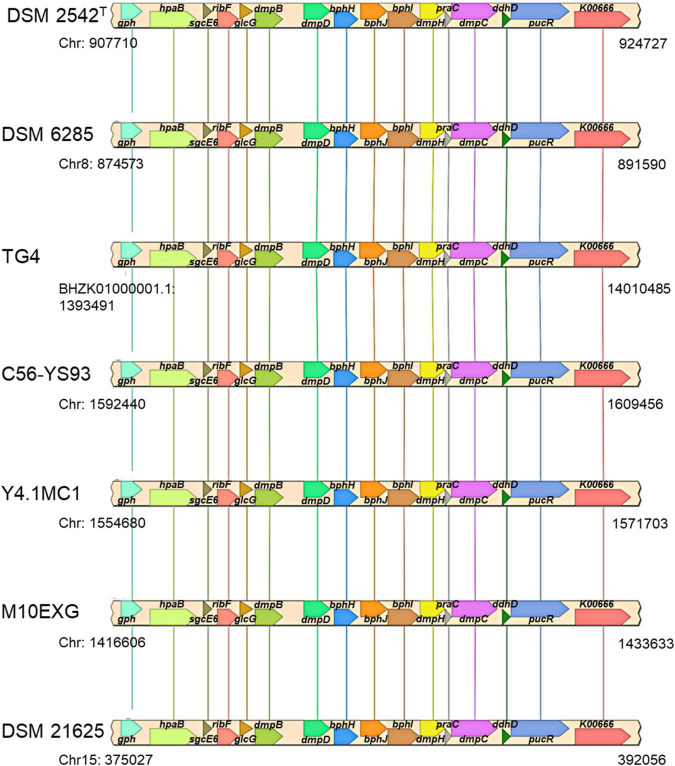
Organization of a chromosomal phenol degradation locus (PtPL) shared by all *P. thermoglucosidasius* strains. *K00666, pucR, ddhD, dmpC, praC, dmpH, bphI, bphJ, bphH, dmpD, dmpB, glcG, ribF, sgcE6, hpaB*, and *gph* code for fatty-acyl-CoA synthase, purine catabolism regulatory protein, CDP-4-dehydro-6-deoxyglucose reductase, 3, aminomuconate-semialdehyde, 4-oxalocrotonate tautomerase, 2-oxo-3-hexenedioate decarboxylase, 4-hydroxy-2-oxovalerate/4-hydroxy-2-oxohexanoate aldolase, acetaldehyde/propanal dehydrogenase, 2-oxopent-4-enoate/*cis-*2-oxohex-4-enoate hydratase, 2-hydroxymuconate-semialdehyde hydrolase, catechol 2,3-dioxygenase, glc operon protein, riboflavin kinase/FMN adenylyltransferase, flavin reductase, 4-hydroxyphenylacetate 3-monooxygenase, and phosphoglycolate phosphatase.

The plasmid-borne phenol catabolic HSUL 2 locus discussed above and the ability to metabolize phenol have been previously reported in *P. thermoglucosidasius* DSM 6285 (formerly reported as *G. stearothermophilus* DSM 6285) (Omokoko et al., 2008). However, the study could not identify *dmpD* and *dmpC* (Omokoko et al., 2008). The latter gene, however, is harbored by all *P. thermoglucosidasius* strains in a chromosomal phenol degradation locus (Duffner et al., 2000) (hereafter referred to as *P. thermoglucosidasius* phenol degradation locus PtPL) located between 907,961 and 924,476 bp on the chromosome of *P. thermoglucosidasius* DSM 2542^T^ (Figure 8). Furthermore, eight of the proteins (BphH-J, DmpB, D and H, PraC and SsgcE6) encoded on the PtPL locus share 58.4% (range: 40.6–73.2%) sequence identity among the respective orthologs in all strains with the corresponding proteins coded by HSUL 2 genes. The PtPL locus, previously characterized in *P. thermoglucosidasius* A7 (Duffner et al., 2000; Kirchner et al., 2003), harbors two genes, *sgcE6* and *hpaB* (Figure 8), which code for a flavin reductase and a 4-hydroxyphenylacetate 3-monooxygenase, respectively. SgcE6 and HpaB from *P. thermoglucosidasius* DSM 2542^T^, share 91.93 and 97.21% identity with PheA1 (AAF66547.1) and PheA2 (AAF66546.1) of *P*. *thermoglucosidasius* A7. The two proteins have been proposed to form a two-component phenol hydroxylase (PheA) which catalyzes the oxidation of phenol to catechol (Duffner et al., 2000; Xun and Sandvik, 2000; Dresen et al., 2010).

In addition to disparate gene order, PtPL and HSUL 2 differ by the incorporation of *dmpC* which codes for an aminomuconate-semialdehyde dehydrogenase, and *petT* which codes for a [2Fe-2S] ferredoxin, respectively (Figures 6, 8). DmpC catalyzes the first step of the 4-oxalocrotonate route of catechol meta-cleavage (Figure 7) where 2-hydroxymuconate-semialdehyde is first converted to 2-oxopent-4-enoate *via* 2-hydroxymuconate and gamma-oxalocrotonate (Díaz and Timmis, 1995; Inoue et al., 1995).

#### RNA-seq Data Suggest Activity of HSUL 1 and 2 During Water Gas Shift Reaction in *Parageobacillus thermoglucosidasius* DSM 6285

**FIGURE 9:**
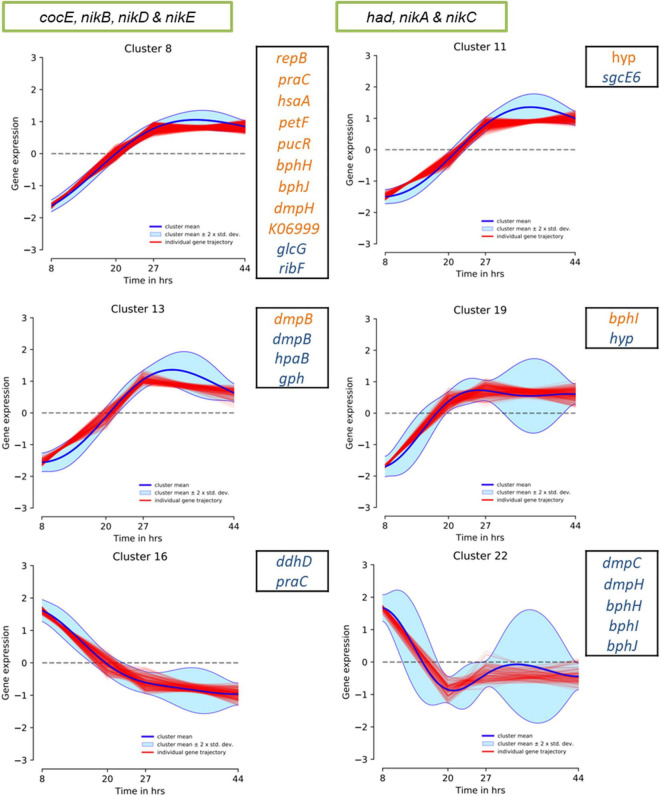
DP_GP_cluster trajectory of *P. thermoglucosidasius* DSM 6285 differentially expressed genes placed in clusters 8, 11, 13 and 19. The strain was cultivated under an initial gas atmosphere of 50% CO and 50% air and samples were taken for expression analysis using RNA-seq over four time points (Aliyu et al., 2020). Green box indicates genes placed in HSUL 1 and black box highlights plasmids genes of HSUL 2 and PtPL. Blue and orange fonts indicate PtPL and HSUL 2 genes, respectively.

To speculate on the potential activity of the above genomic regions under WGS reaction conditions, we evaluated the previously reported *P. thermoglucosidasius* DSM 6285 transcriptome data. Specifically, the data reported the expression profiles of genes showing significant differential gene expression during growth of *P. thermoglucosidasius* DSM 6285 under 50% CO and 50% air atmosphere (Aliyu et al., 2020). The data revealed that *nikABCDE* were among genes that were generally downregulated (clusters 8 and 11) during the initial aerobic growth phase (8 h) in DSM 6285 and overexpressed during the WGS reaction (time points 27–44 h) (Figure 9). All five genes (*nikABCDE*) were significantly (*p* < 0.05) upregulated at 20 h (relative to 8 h) and at 27 h (relative to 20 h) but no significant differential genes expression was observed between 27 and 44 h post-inoculation (Supplementary Table 3).

Evaluation of the HSUL 2 genes revealed that all nine genes showed expression trajectories like the *nik* genes (Figure 9). However, no significant difference was observed in the expression of *bphI* between 20 and 27 h post-inoculation (Supplementary Table 3). Similarly, transcripts of four PtPL genes (*dmpB*, *hpaB*, *gph* and *hyp*) showed the general pattern of increased expression in the latter stages of *P. thermoglucosidasius* DSM 6285 cultivation under an initial gas composition of 50% CO and 50% air (Figure 9). Unlike the plasmid-borne copy of *dmpB*, transcripts of its chromosomal orthologs did not show significant increase between 20 h and 27 h post-inoculation (Supplementary Table 3). By contrast, transcripts of seven PtPL genes showed the opposite trend (clusters 16; *ddhD* and *praC* and cluster 22; *dmpC*, *dmpH*, *bphH*-j; Figure 9), with overexpression during the initial aerobic growth phase and downregulation over the WGS reaction phase (Figure 9). However, no significant differences in gene expression was observed between the late aerobic growth phase (20 h) and the later stages of the cultivation (27 and 44 h) for transcripts of cluster 22 while transcripts of *ddhD* and *praC* were significantly depleted at 27 and 44 h post-inoculation (Supplementary Table 3).

## Discussion

Cultivation experiments with *P. thermoglucosidasius* strains that have not been previously characterized for WGS reaction showed that the ability to oxidize CO is highly restricted in this species. Of the eleven *P. thermoglucosidasius* strains so far cultivated on CO, including the seven reported in this study, seven (∼ 63%) strains lack the capacity to catalyze the WGS reaction. Based on the reconstructed *gyrA-recN-rpoB* and SCOs phylogenies it is deducible that the genetic events associated with this phenotype occurred independently in several lines after the acquisition of the genetic elements conferring the WGS reaction phenotype by the common ancestor of the strains. For instance, both indel 1 and 2 occur only in the *cooCSF-phcA-L* regions of *P. thermoglucosidasius* DSM 21625 and C56-YS93 and only indel 1 and indel 2 are present in *P. thermoglucosidasius* Y4.1MC1 and Kp1012, respectively. However, the latter strain appears to incorporate a distinct genetic element in the position of indel 1, which suggest a possible progression of these evolution events in Kp1012 and perhaps other members of the species. These indels are however, absent in the *P. thermoglucosidasius* Kp1019, M10EXG and M5EXG, which nevertheless do not catalyze WGS reaction like the above strains.

Previous evaluation of the genetic region of the above deletions identified only a single TFB site for the transcriptional regulator Hpr (Mohr et al., 2018b), which is associated with regulation of several functions, such as antibiotic production, motility, and sporulation, upstream of indel 1 (Kallio et al., 1991; Mohr et al., 2018b). The current analysis, however, showed that although the genomic region surrounding these indels appears to contain several TFBs, only the TFB site of the aerobic respiration control protein ArcA is directly within the genetic elements in indel 2. ArcA regulates the ArcAB system linked to low O_2_ (microaerobic) induced regulation of central metabolic pathways, including the suppression of TCA cycle and respiratory chains enzymes and induction of fermentative pathways in *E. coli* (Alexeeva et al., 2003; Nochino et al., 2020). The ArcAB system is also associated with resistance against reactive oxygen species during aerobic growth in bacteria (Loui et al., 2009). While it is plausible that the ArcAB system is involved in modulating the WGS reaction, the genomic regions adjacent to these indels harbor several alternative putative ArcA binding sites (Table 2). Further complicating such speculation is the absence of both indel 1 and 2 in three strains incapable of hydrogen production under the above conditions.

Unlike the *cooCSF-phcA-L* locus-associated deletions, our analysis revealed that genes coding for proteins involved in nickel and iron binding and transport are consistently present in genomes of strains with proven WGS reaction capability and missing in the strains without the phenotype. Nickel is an essential component of the WGS reaction enzymes, forming part of the metallocentre of both CODH and Phc (Eitinger and Mandrand-Berthelot, 2000; Mohr et al., 2018a,b). The absence of the putative nickel transporter (*nikABCDE*) locus could hinder nickel uptake and hence the function of these enzymes. In *E*. *coli*, it was demonstrated by mutagenesis studies that the nickel transporter operon was essential for nickel uptake and Ni-Fe hydrogenase activity (Cavazza et al., 2011). Similarly, hydrogenase activity was completely suppressed with the deletion of the *nikA* homolog *nikZ* in the *nik* operon of *Campylobacter jejuni* (Howlett et al., 2012). Our temporal RNA-seq study spanning the WGS reaction phase further showed that the putative nickel transport genes were among 1,317 genes downregulated during the initial aerobic growth and significantly upregulated during the anaerobic growth phase, including the WGS reaction phase (Aliyu et al., 2020). In addition to CODH and hydrogenases, which are associated with WGS reaction, nickel availability could influence the activity of other *P. thermoglucosidasius* nickel-containing enzymes. For instance, acireductone dioxygenase (WP_003252143.1) and urases (e.g., WP_003251018.1), involved in methionine salvage pathway and hydrolysis of urea, respectively, use nickel as a co-factor (Boer et al., 2014). Likewise, the potential nickel uptake role of other *P. thermoglucosidasius* predicted ABC transporters with substrate binding modules (e.g., WP_042385428.1) and associated permease proteins (e.g., WP_003252829.1) is subject for further investigation.

The second unique set of genes, which demonstrate an expression trajectory similar to the nickel transport genes, occur on the plasmid of the *P. thermoglucosidasius* DSM 2542^T^ and the other hydrogenogenic *P. thermoglucosidasius* strains. The plasmid region incorporates genes that encode enzymes which catalyze the complete module for the degradation of phenol to acetyl-CoA *via* the catechol meta-cleavage pathway (Phale et al., 2019). In *B. subtilis*, the synthesis of catechol 2,3-dioxygenase (DmpB), a major enzyme in this pathway, was reported to be induced under iron limitation in anticipation of the accumulation of catechol by-product of ferric-bacillibactin complex hydrolysis (Pi et al., 2018). Hydrolysis of ferric-bacillibactin by ferric-bacillibactin esterase BesA releases iron, thereby making it available for incorporation into the metallocentres of several enzymes (Mortensen and Skaar, 2013; Harwood et al., 2018; Pi et al., 2018).

Although siderophore-dependent iron acquisition is well known among bacteria (Raymond et al., 2003; Miethke et al., 2011), this system has not been demonstrated in *P. thermoglucosidasius*. However, *P. thermoglucosidasius* genomes harbor putative genes that code for iron-siderophore transport system ATP-binding protein (e.g., WP_003249888.1) and iron-siderophore transport system permease proteins (e.g., WP_003249886.1 and WP_003249886.1). Similarly, *P. thermoglucosidasius* harbor genes which code for proteins involved in siderophore biosynthesis. For instance, the dimodular non-ribosomal peptide synthetase (DhbF) from *P. thermoglucosidasius* Y4.1MC1 was used to demonstrate the organization of non-ribosomal peptide synthetases (NRPS), associated with synthesis of siderophore (Felnagle et al., 2008; Tarry et al., 2017). Moreover, CO induced iron starvation and the corresponding increased synthesis of siderophores have been demonstrated in bacteria (Wareham et al., 2016).

Of note, both the plasmid (HSUL 2) and *P. thermoglucosidasius* chromosome harbor genes that code for enzymes associated with degradation of aromatic compounds, including phenol (and catechol). Previous studies have reported the ability of *P. thermoglucosidasius* strain to catabolize phenol, putatively *via* the action of enzymes encoded on either of the plasmid and chromosomal loci (Duffner et al., 2000; Kirchner et al., 2003; Omokoko et al., 2008). However, transcriptome analysis with *P. thermoglucosidasius* DSM 6285 showed an increased genetic activity in the HSUL 2 locus over the transition between aerobic growth and WGS reaction phase. At the same time, expression of PtPL genes associated with metabolism of the by-product of catechol cleavage (*dmpCH* and *bphH-J*) was suppressed. The significance of the differences observed in the expression of phenol catabolic genes of the two loci is a subject for further investigation. It could be speculated, however, The HSUL2 locus gene *petF*, which codes for a 2Fe-2S ferredoxin and is unique to the hydrogenogenic *P. thermoglucosidasius* strains, plays a central role in modulating catechol degradation over the period prior to WGS reaction. Previous studies have demonstrated the role of PetF in stabilizing or reactivating DmpB by reducing the iron atom in the active site of the enzyme to its ferrous state (Polissi and Harayama, 1993; Hugo et al., 1998; Armengaud et al., 2000).

In conclusion, this study revealed that the majority of the *P. thermoglucosidasius* strains hitherto analyzed are unable to catalyze the WGS reaction under the described conditions. Apparent gene loss events, involving genes linked to intracellular detoxification as well as nickel and iron availability, likely play a central role in shaping the observed differences in the hydrogenogenic abilities of *P. thermoglucosidasius* strains. However, these do not preclude the ability of the non-CO oxidizers to catalyze the WGS reaction under modified cultivation conditions, for instance supplementation with optimal amounts of nickel and iron. Further characterization of both nickel and iron uptake systems of *P. thermoglucosidasius* may provide clearer insight into their roles in modulating WGS reaction.

## Data Availability Statement

The original contributions presented in the study are included in the article/Supplementary Material, further inquiries can be directed to the corresponding author/s.

## Author Contributions

HA, AN, and PM conceived the study and edited the manuscript. HA and AN designed the experiments. HA conducted the experiments, genomic analyses, and wrote the initial manuscript. All authors read and approved the final version of the manuscript.

## Conflict of Interest

The authors declare that the research was conducted in the absence of any commercial or financial relationships that could be construed as a potential conflict of interest.

## Publisher’s Note

All claims expressed in this article are solely those of the authors and do not necessarily represent those of their affiliated organizations, or those of the publisher, the editors and the reviewers. Any product that may be evaluated in this article, or claim that may be made by its manufacturer, is not guaranteed or endorsed by the publisher.
