# Supplementary material for: Not All That Glitters Is Gold: The Paradox of CO-dependent Hydrogenogenesis in Parageobacillus thermoglucosidasius
Source: Front Microbiol. 2021 Dec 9;12:784652. doi: 10.3389/fmicb.2021.784652 (PMC8696081; doi:10.3389/fmicb.2021.784652)
Supplement: Supplementary file 1 [file Data_Sheet_1.zip › Figure S1 - S2.DOCX]

Supplementary Material

## Supplementary Figures

**
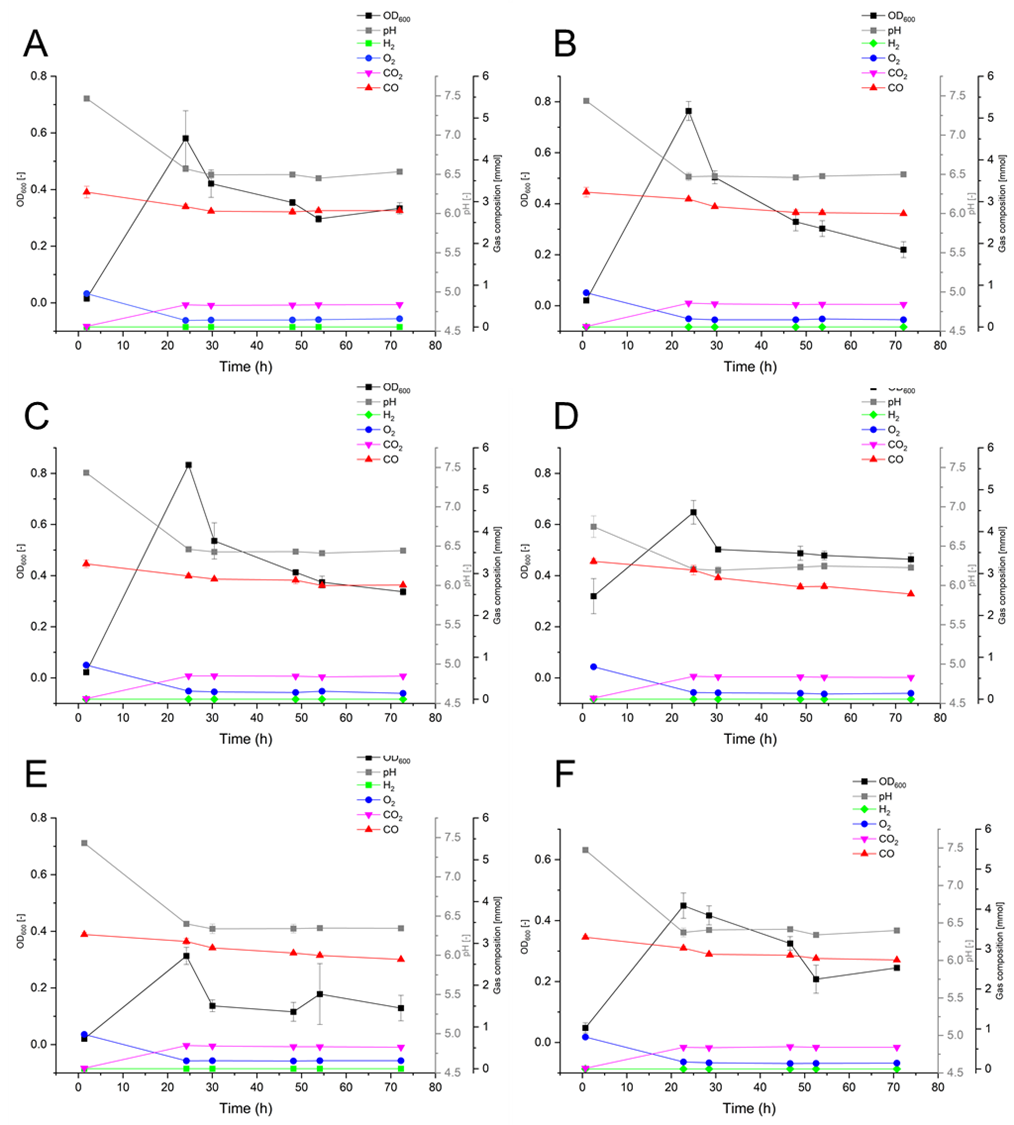
**

Supplementary Figure S1: Gas composition and growth of non-hydogenogenic *P. thermoglucosidasius* strain. (A) C56-YS93, (B) M5EXG, (C) M10EXG, (D) Kp1012, (E) Kp1019, and (F) Y4.1MC1, cultivated under 50% carbon monoxide and 50% air in 250 ml serum bottles containing 50 ml of mLB medium.


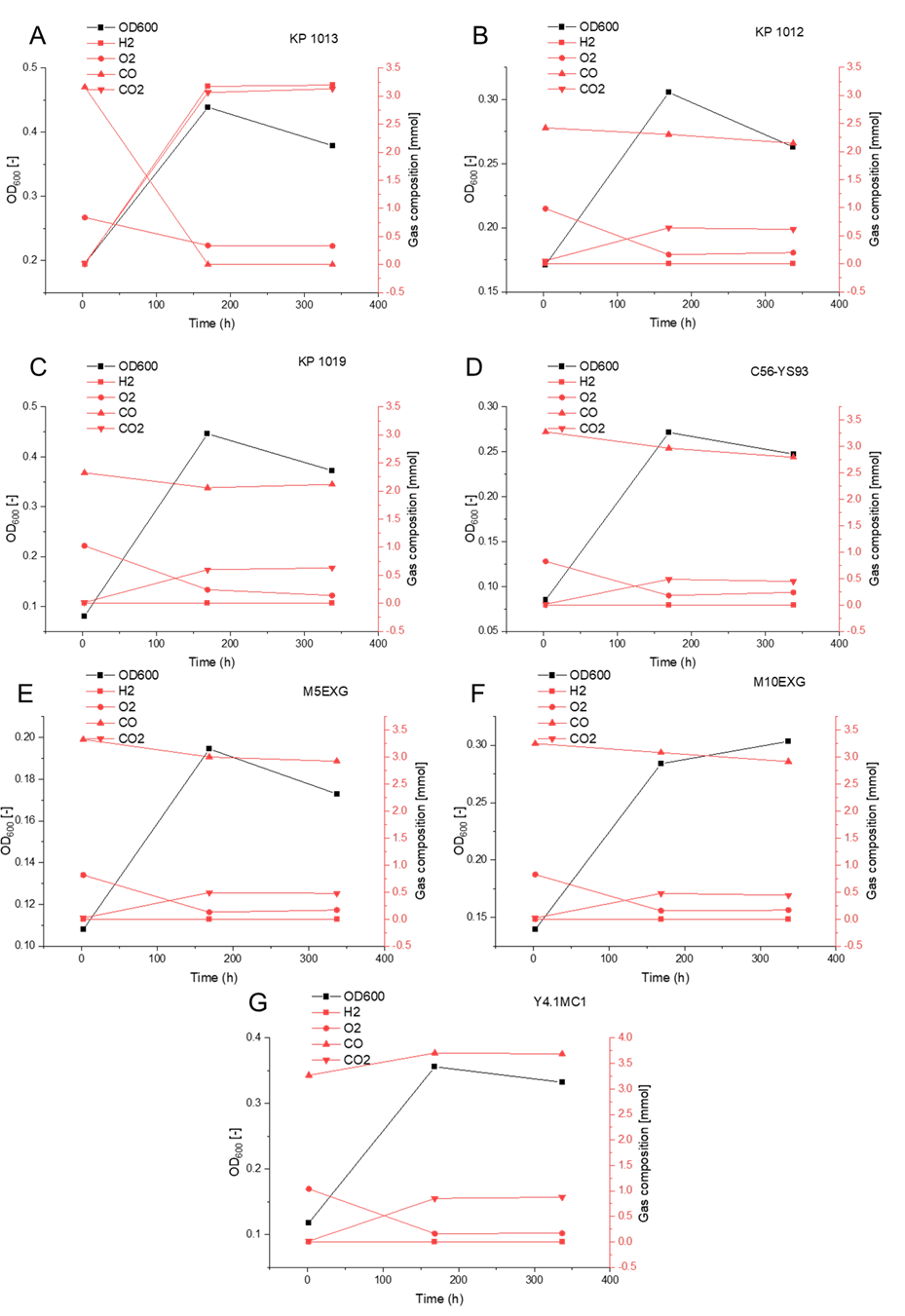


**Supplementary Figure S2: Gas composition and growth of *P. thermoglucosidasius* strain cultivated under 50% carbon monoxide and 50% air in 250 ml serum bottles containing 50 ml ASM medium. (A) Kp 1013, (B) Kp1012, (C) Kp1019, (D) C56-YS93, (E) M5EXG, (F) M10EXG, (D), and (G) Y4.1MC1. Strains were cultivated in duplicates and samples collected three times over 337 hours.**
